# Selling Misleading “Cancer Cure” Books on Amazon: Systematic Search on Amazon.com and Thematic Analysis

**DOI:** 10.2196/56354

**Published:** 2024-10-08

**Authors:** Marco Zenone, May van Schalkwyk, Greg Hartwell, Timothy Caulfield, Nason Maani

**Affiliations:** 1 Faculty of Public Health and Policy London School of Hygiene & Tropical Medicine London United Kingdom; 2 Health Law Institute Faculty of Law University of Alberta Edmonton, AB Canada; 3 Global Health Policy Unit The University of Edinburgh Edinburgh United Kingdom

**Keywords:** cancer, Amazon, misinformation, e-commerce, cancer cure, cancer misinformation, misleading, cancer information, treatment, cancer treatment, thematic analysis, misleading, online information

## Abstract

**Background:**

While the evidence base on web-based cancer misinformation continues to develop, relatively little is known about the extent of such information on the world’s largest e-commerce website, Amazon. Multiple media reports indicate that Amazon may host on its platform questionable cancer-related products for sale, such as books on purported cancer cures. This context suggests an urgent need to evaluate Amazon.com for cancer misinformation.

**Objective:**

This study sought to (1) examine to what extent are misleading cancer cure books for sale on Amazon.com and (2) determine how cancer cure books on Amazon.com provide misleading cancer information.

**Methods:**

We searched “cancer cure” on Amazon.com and retrieved the top 1000 English-language book search results. We reviewed the books’ descriptions and titles to determine whether the books provided misleading cancer cure or treatment information. We considered a book to be misleading if it suggested scientifically unsupported cancer treatment approaches to cure or meaningfully treat cancer. Among books coded as misleading, we conducted an inductive latent thematic analysis to determine the informational value the books sought to offer.

**Results:**

Nearly half (494/1000, 49.4%) of the sampled “cancer cure” books for sale on Amazon.com appeared to contain misleading cancer treatment and cure information. Overall, 17 (51.5%) out of 33 Amazon.com results pages had 50% or more of the books coded as misleading. The first search result page had the highest percentage of misleading books (23/33, 69.7%). Misleading books (n=494) contained eight themes: (1) claims of efficacious cancer cure strategies (n=451, 91.3%), (2) oversimplifying cancer and cancer treatment (n=194, 39.3%), (3) falsely justifying ineffective treatments as science based (n=189, 38.3%), (4) discrediting conventional cancer treatments (n=169, 34.2%), (5) finding the true cause of cancer (n=133, 26.9%), (6) homogenizing cancer (n=132, 26.7%), (7) discovery of new cancer treatments (n=119, 24.1%), and (8) cancer cure suppression (n=82, 16.6%).

**Conclusions:**

The results demonstrate that misleading cancer cure books are for sale, visible, and prevalent on Amazon.com, with prominence in initial search hits. These misleading books for sale on Amazon can be conceived of as forming part of a wider, cross-platform, web-based information environment in which misleading cancer cures are often given prominence. Our results suggest that greater enforcement is needed from Amazon and that cancer-focused organizations should engage in preemptive misinformation debunking.

## Introduction

Following a cancer diagnosis, patients with cancer understandably turn to web-based sources to look for information on treatment, to seek emotional support [1], or to learn from the experiences of other patients with cancer [2]. Unfortunately, the web-based informational cancer environment they navigate is often polluted with inaccurate and harmful cancer information. A recent survey of patients with cancer found that over 55.9% reported seeing cancer misinformation on the web and 25% received advice on alternative cancer cures [3], which are associated with worse outcomes [4,5]. Cancer treatment misinformation is prevalent on social media platforms [6] such as YouTube [7,8], TikTok [9], Facebook [10], Twitter (rebranded as X) [11], and Pinterest [12] and has been found to be amplified by search algorithms [13]. Multiple studies suggest that misleading cancer information receives more engagement than factual sources [6,14] and that cancer misinformation is partially driven by financial interests and incentives [15,16]. Web-based cancer misinformation unfairly burdens patients with cancer and those close to them, who are forced to evaluate and navigate complex, “believable” yet false cancer information at a time of emotional and psychological strain [17].

Amazon is the world’s largest e-commerce website. Each day, tens of thousands of products are sold on the platform to Amazon’s 300+ million users [18]. Amazon assists its users to sell, advertise, or purchase products in an efficient and convenient process with few barriers [19]. As of October 2023, a search for “cancer” on Amazon (Amazon.com) returns over 100,000 search results [20]. Amazon purports to restrict what types of products can be sold to protect its users from deceptive advertising or products and comply with the laws and regulations of the countries in which it operates. For example, Amazon prohibits product claims for any disease that do not have the approval of the US Food and Drug Administration (FDA) [21]. This policy would likely disallow any unapproved products stating they can cure, prevent, or meaningfully treat cancer.

However, in spite of such policies existing, multiple media reports document sellers on Amazon listing dangerous and “misleading” [22] cancer products. Audits have found Amazon to promote antivaccination books [23]. Media reporting suggests that Amazon hosted COVID-19 conspiracy documentaries [24] and other COVID-19 misinformation [25]. Related to cancer, it has been suggested that Amazon gives recommendations for books advising unproven or disproven cancer cures [26,27]. At the time of writing, the number one best seller under “cancer” on Amazon is a book that describes how a person with cancer healed himself naturally after refusing chemotherapy following surgery [28]. Media reports note that the book presents a misleading narrative, in which the author opted out of curative chemotherapy when he in fact opted out of adjuvant chemotherapy [29,30]. The author of the book has been demonetized on YouTube [31] but still appears to earn commissions and sell cancer-related books, anticancer supplements, and immune support products on Amazon [32].

This context suggests an immediate need to evaluate Amazon for cancer misinformation. The tools offered by Amazon to its sellers may enable the sale of books promoting scientifically unsupported cancer treatments and cures. Evaluating alleged cancer cure books for sale on Amazon is important to determine the information patients with cancer may view and purchase. Misleading cancer cure information may lead to dangerous treatment decisions [33] and undermine patients’ confidence in evidence-based treatments. Therefore, this study sought to (1) examine to what extent are misleading cancer cure books for sale on Amazon.com and (2) determine how cancer cure books on Amazon.com provide misleading cancer information. The results can provide valuable information to public health authorities and external regulators, to ensure that patients with cancer are not financially exploited to purchase books with harmful information.

## Methods

### Data Collection

We searched “cancer cure” on Amazon.com (10,000+ search results) and retrieved the top 1000 English-language book search results, including sponsored books, on August 3, 2023. Similar to other studies, we performed the search without logging into the platform to avoid the search biases of an existing profile [34]. The Amazon.com search function matches customers to products that fit their typed description. Our search mimics the searches of patients with cancer who may be seeking cancer cure information on Amazon.com. To collect the data, MZ created 2 Data Miner scrapers [35] that retrieved the product information of each result, including its title, author, description, rating score, number of ratings, price, year published, number of pages, publisher-supplied “about the author” information, and the page number of the Amazon.com search result that the book appeared in. Data Miner is a program that allows its user to create simple scrapers that automatically collect certain fields from a web page. The first Data Miner scraper collected the URLs of each result listed from the “cancer cure” Amazon.com search. The second Data Miner scraper was created to collect the aforementioned web page fields (eg, book name) from each of the URLs collected from the first scrape.

### Designating a Book as Misleading

MZ reviewed the books’ descriptions and titles to determine whether the books provided misleading cancer cure or treatment information. As the books are for sale and their titles and descriptions represent their value to a potential customer, we considered this information appropriate to determine whether the books for sale are misleading or not. We considered a book to be misleading if it suggested scientifically unsupported cancer treatment approaches that cure or meaningfully treat cancer. To determine which books are misleading, we consulted a list of alternative and natural treatments named in a study examining which alternative cancer treatments patients use [36], along with the National Cancer Institute’s list of alternative and complementary cancer treatments [37]. While it is not possible to identify every unproven cancer cure, our sources contain the standard and most well-known examples. We then applied the lists against the results. We additionally reviewed books for misleading cues. This included overt statements that are contrary to scientific or clinical consensus and present conspiratorial narratives. To ensure consistent inclusion decisions, TC blindly audited 10% of inclusion decisions. TC found no disagreements, demonstrating high agreement and consistent coding. TC additionally reviewed the code applications MZ marked as requiring a second opinion.

### Analysis

We conducted a qualitative, inductive latent thematic analysis [38] to determine the informational value the books sought to offer to potential customers. We followed the thematic analysis steps outlined by Clarke and Braun [39], consisting of data familiarization, generation of initial codes, searching for themes, reviewing themes, defining and naming themes, and reporting. To increase the trustworthiness of the analysis, the authors adopted the recommendations of Nowell et al [40]. MZ, GH, TC, and NM reviewed the data, undertook data review, and iteratively drafted and redrafted a coding frame until a final frame captured themes. MZ coded the data. GH audited nearly half (236/494, 47.8%) of thematic book coding, finding agreement with 95.8% (1,808/1,888) of code decisions, signifying consistent and accurate coding. All disagreements were resolved through discussion. GH reviewed the thematic code applications MZ marked as requiring a second opinion. Our study followed the Standards for Qualitative Research Reporting guidelines (Multimedia Appendix 1) [41].

### Ethical Considerations

This study did not require ethical approval because all data collected and analyzed are publicly available and posted without the expectation of privacy.

## Results

### Frequency of Misleading Cancer Cure Books

**Figure 1:**
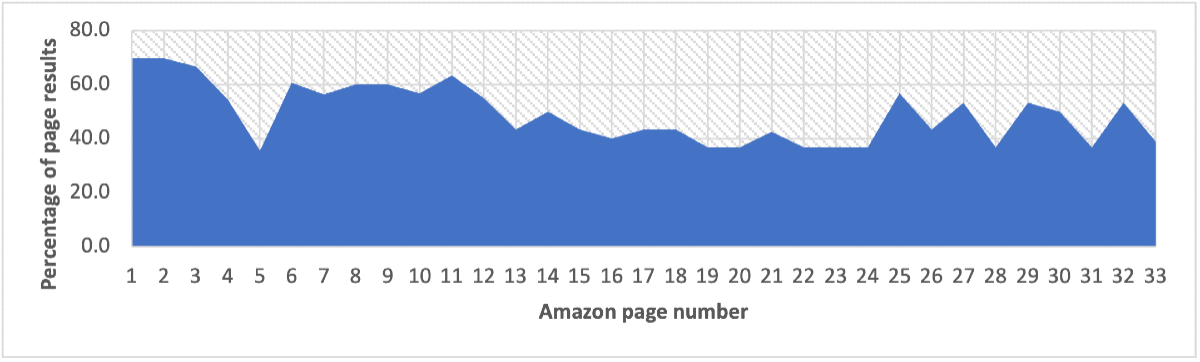
Percentage of misleading cancer books on Amazon.com by search result page number.

Our analysis found that 49.4% (494/1000) of the sampled “cancer cure” books for sale on Amazon.com contained misleading cancer treatment or cure information. Misleading books were consistent throughout the search result pages (Figure 1), with 17 (51.5%) out of 33 Amazon.com results pages having 50% or more of the books coded as misleading. Results in the first 3 pages contained the highest percentage of misleading books (first page: 23/33, 69.7%; second page: 23/33 69.7%; third page: 22/33, 66.7%). The lowest percentage of misleading books across any page found was 35.5% (11/31).

### Misleading Cancer Book Themes

#### Overview

Misleading books (n=494) contained eight themes: (1) claims of efficacious cancer cure strategies (n=451, 91.3%), (2) oversimplifying cancer and cancer treatment (n=194, 39.3%), (3) falsely justifying ineffective treatments as science based (n=189, 38.3%), (4) discrediting conventional cancer treatments (n=169, 34.2%), (5) finding the true cause of cancer (n=133, 26.9%), (6) homogenizing cancer (n=132, 26.7%), (7) discovery of new cancer treatments ( n=119, 24.1%), and (8) cancer cure suppression (n=82, 16.6%). Their frequencies and definitions are shown in Table 1.

**Table 1:** Misleading cancer books themes by frequency and illustrative example.

Theme	Frequency (n=494), n (%)	Definition
Claims of efficacious cancer cure strategies	451 (91.3)	Treatment can or has cured cancer
Oversimplifying cancer and cancer treatment	194 (39.3)	Cancer is not complicated and is simple to understand, treat, and cure. Cancer can be treated using simple foods or at-home remedies
Falsely justifying ineffective treatments as science based	189 (38.3)	Ineffective cancer cures are scientifically proven to work Justified by the author’s own academic research Contains unsupported conclusions or demonstrates a lack of understanding of scientific processes and evidence thresholds
Discrediting conventional cancer treatments	169 (34.2)	Critical descriptions of chemotherapy, radiation, surgery, “Western medicine,” and conventional treatments, including side effects, causing harm (poisoning, worsening cancer, and killing patients with cancer), and lack of effectiveness
Finding the true cause of cancer	133 (26.9)	Treating cancer requires understanding the root cause of cause, why cancer occurs, and presentations of paradigm shifts on what’s known to cause cancer
Homogenizing cancer	132 (26.7)	One treatment can cure all types of cancer and/or other diseases
Discovery of new cancer treatments	119 (24.1)	People with cancer, their family, or health practitioners finding new cancer cures and treatment options
Cancer cure suppression	82 (16.6)	Cancer cures already exist but are hidden or banned because of financial interests from the pharmaceutical industry, legal battles with regulators or medical associations, and conspiracies to ruin reputations

#### Theme 1: Claims of Efficacious Cancer Cure Strategies

The most observed theme, claims of efficacious cancer cure strategies (n=451, 91.3%), referred to books giving cancer treatment information that is alleged to cure or meaningfully treat cancer. Here, language that directly posits efficacy for cancer treatment is used. For example, a cookbook with a title indicating that a specific diet can cure cancer states the following: “Approved, tested, and trusted recipes to reverse, prevent and cure cancer diseases completely.” These books provided efficacy assurances demonstrating supposed effectiveness.

#### Theme 2: Oversimplifying Cancer and Cancer Treatment

How to treat, cure, and think about cancer was oversimplified in 194 (39.3%) books. Books claimed that cancer is not complicated to treat but rather is simple to understand, treat, and cure. Books suggested simple remedies capable of curing cancer. For example, a book description states that it is possible to cure cancer solely using carrot juice: “In 2013, [anonymized] cured stage 4 colon cancer without chemotherapy or radiation by drinking carrot juice…[anonymized] wanted to find out why such an apparently simple cancer cure – just carrots – works.” Books also provided unrealistic timelines for how long simple cures or treatment approaches would take to cure cancer. For example, another book states that it is possible to cure cancer within a week to a month using anticancer remedies: “The ultimate cure for bone cancer is here, and it is not as complicated to be found as we have been made to think…cure your cancer with anticancer remedies within a month or maybe within a week.” Books simplifying cancer present an inaccurate portrayal of complex diseases and treatment decisions, including consideration of a person’s comorbidities and current medications.

#### Theme 3: Falsely Justifying Ineffective Treatments as Science Based

Relatedly, books used scientific-sounding language to justify that their outlined treatments are suitable and efficacious for primary cancer treatment (n=189, 38.3%). Authors described their own research and presented unsupported conclusions that demonstrate a lack of understanding or appreciation of the scientific process and evidence standards. For example, a book with a title indicating that their advocated cancer therapy is scientifically proven states the following: “The [anonymized book title] reveals a scientific, clinically proven natural therapy for healing cancer and creating optimal health.” The misuse of scientific language and standards inaccurately portrays misleading treatment as being effective for cancer and misrepresents established scientific consensuses.

#### Theme 4: Discrediting Conventional Cancer Treatments

Perceived inadequacies of scientifically supported cancer treatments and alternatives to them were found in 169 (34.2%) results. Books provided critical descriptions of chemotherapy, radiation, surgery, “Western medicine,” and other conventional cancer treatments, including their side effects; unsupported statements on the lack of effectiveness; and allegations that they cause unjustifiable harms such as poisoning, worsening of cancer, and death. For example, a book promoting alternative cancer treatments states the following: “cancer patients are still given questionable chemotherapies, which are proven to have no effect at all in the case of most cancer…why are cancer patients treated with a healing method that triggers this disease.” Books represented their treatments as filling the gaps of inadequate cancer care. For example, a book description states that “death is blamed on cancer [after] chemotherapy’ rather than deception and inappropriate treatments…” Books alleging the inadequacies of conventional cancer treatments disparage best treatment practices and undermine health providers.

#### Theme 5: Finding the True Cause of Cancer

Books argued that curing cancer requires an understanding of the true cause of cancer and why cancer occurs (n=133, 26.9%). At times, books linked to previously established risk factors for cancers, such as processed foods, but misleading books suggested oversimplified, exaggerated, or unsupported reasons why cancer develops. For example, a book suggests that cancer may be a fungus or that cancer is caused by fungi: “It’s been said that cancer is a fungus…while the verdict is still out cancer is a fungus, here is indisputable proof that some common fungi, found in foods and certain pharmaceuticals, cause cancer.”

#### Theme 6: Homogenizing Cancer

The homogenization of cancer occurred in 132 (26.7%) books. One treatment was represented to cure all types of cancer and/or other diseases. For example, a book description states the following: “One of the key conclusions that we reached through our individual journeys was that whether you are a sufferer of superficial urinary bladder cancer, or any other condition, the same protocol that we used to heal will apply to you.” In addition to homogenizing cancer types, homogenization of the cause of cancer and other illnesses also occurred. For example, a book giving advice on how to reverse breast cancer through kidney filtration illustrated both this and theme 5 together when stating the following: “We used the same protocols irrespective of condition name as we had found the root cause of all conditions/disease to be the same.” Homogenizing cancer treatments for all cancers or other diseases provides misleading information and ignores the complexities of cancer care.

#### Theme 7: Discovery of New Cancer Treatments

Discovery of and creation of new alleged cancer cures were seen in 119 (24.1%) books. Here, authors described how they or the person they’re writing about developed or found a new cancer treatment protocol. The motivation for the discovery was typically due to a personal situation, such as the author themselves or a family member having an incurable form of cancer, or a medical provider or scientist committing to finding a new cure. For example, a book telling the story of a man who found a tea that cures cancer states the following: “This is the extraordinary account of the cancer remission of a man told he would be dead in two or three months, what he decided to do, and the almost miraculous tea that he accidentally created, which paved the way for his cancer’s disappearance, never to return.” Cancer treatment discovery books provide narratives of persons in desperate situations that may be compelling for readers facing similar challenges.

#### Theme 8: Cancer Cure Suppression

The last theme, cancer cure suppression, refers to claims that cancer cures already exist but are suppressed due to the vested interests of various others (n=82, 16.6%). These books argue that they are hidden or banned from practice because of financial interests from the pharmaceutical industry, purposeful legal battles with regulators and medical associations, and conspiracies to ruin personal reputations. For example, a book description states the following: “This book exposes the cures for cancer that are discovered and then suppressed by corporate and political interests. Big medical companies are dependent on a continuing increase in cancer so that they can continue increasing their profits.” Books also allege that certain figures, sometimes the author themselves or another public figure, are being cancelled or censored. For example, a book description states: the following “His [anonymized name] internet speech was banned by a federal judge in 2014 on behalf of the FDA in order to hide natural cures from the public.” Books alleging that cancer cures are suppressed promote conspiracy theories and revise or misrepresent historical events and decisions.

## Discussion

### Principal Findings

These results demonstrate that misleading cancer cure and treatment books are for sale, visible, and seemingly prevalent on Amazon.com. Nearly half of the “cancer cure” books for sale offered misleading and potentially harmful cancer treatment misinformation. Misleading books directly claimed to have efficacious cures for cancer, undermined scientifically supported treatments, misapplied scientific reasoning, oversimplified cancer and cancer treatments, and promoted conspiracy theories. Books offered information that may delay or encourage patients to opt out of best-standard treatments and create false hope.

Our study contributes to the increasing research documenting the presence, spread, and mechanisms of medical misinformation on Amazon. Notably, Amazon’s algorithm has been reported to amplify books with vaccine misinformation [23,42]. In other cases, Amazon has been found to host products selling fake autism cures [43] and promote COVID-19 misinformation [25]. Amazon’s search results have been found to rank products with misinformation higher than sources debunking misinformation [34]. Concerningly, misinformation in Amazon-hosted products may be amplified by fake product reviews, which are associated with a causal increase in product sales [44,45].

Amazon effectively gives legitimacy affordances [46] to authors without medical credentials to freely write medical information and advice books. Previous studies have documented how other technology platforms, like Meta and Google, may inadvertently enable persons selling disproven or unproven cancer therapies to appear to be legitimate medical providers [10]. Many books in our study were self-published, thus undergoing limited or no external or publisher review [22]. Amazon may enable such books to be put up for sale, advertised, matched to user search queries depending on keywords, and displayed alongside books written by qualified authors. The blurred differentiation between books offering misleading or credible information creates unfair difficulties for patients with cancer evaluating sources of web-based information during a highly stressful time in their lives.

The presence of misleading cancer cure books for sale on Amazon occurs despite Amazon policies that prohibit non–FDA-approved medical claims [21]. Presumably, this policy would include books whose market value is derived from the cancer treatment information they offer, although this is not immediately clear. The findings of the study may support Amazon to expand or revise its existing policies to protect its users. The presence of misleading cancer cure books also occurs after previous criticism of Amazon for allowing the sale of books with misleading information during the COVID-19 pandemic [47,48]. This context suggests that Amazon’s disinformation and misinformation policies related to the sale of health-related books are at this time limited, inadequately protecting its users.

Amazon benefits from a distinct form of “platform power” [49] that complicates regulators’ abilities and their political support to act upon health misinformation. Platform power refers to the “tacit allegiance” of a platform’s consumers. Confronting the mechanisms that make Amazon successful, such as its algorithms, affordances to sellers, advertisements, and convenience, is likely not in the interest—and would feel beyond the power—of many Amazon users. This also limits the public support of political entities to act upon health misinformation on Amazon. Therefore, while receiving this distinct platform power, Amazon, like other big tech platforms, may not be incentivized [50] to act upon health misinformation until its users demand such actions or until its business interests are threatened. It has been argued that digital platforms, which are reliant on automated, algorithmically driven, paid advertising for income, are themselves important commercial determinants of health [50,51]. Furthermore, they may have an inherent conflict of interest, where meaningfully curbing exploitation might require greater resources committed to oversight and regulatory compliance or reductions in the profitability of certain platform features. In light of their scale, power, and contributions to the wider digital ecosystem, such platforms may have cumulative harmful effects on the spread and consistent appearance of such misinformation. This may also occur in ways that make the independent investigation of effective cancer treatment, by those who may have just received a diagnosis, highly challenging in inequitable ways. Inherent to these issues is the tension between the informational needs of the public and their right to be protected from the harms associated with the spread of misinformation on the one hand, and the interests of corporations like Amazon to maximize profits on the other hand.

Our results and those of other studies documenting harmful web-based ecosystems for patients with cancer suggest that cancer-focused organizations should engage in preemptive treatment misinformation debunking [52]. Dispelling cancer misinformation before patients are exposed to it can result in a form of psychological inoculation against ineffective treatments [53]. Patients with cancer, especially those with terminal prognoses, are inevitably susceptible to exploitive actions from bad actors selling false hope [54]. Web-based technology platforms, such as specific social media platforms, Amazon, or Google, inadvertently assist such bad actors in selling their products [10]. Health care providers and cancer support organizations are well placed to preemptively debunk such content, ensuring a first-line defense against cancer misinformation. Proactive surveillance of cancer treatment misinformation and health literacy initiatives can help curb misinformation effects.

### Limitations

Our study has several important limitations. We collected and analyzed the publicly available information from a book’s Amazon.com listing page. It was not feasible nor ethical to purchase and read the entire book’s content. However, the product information collected, including the book’s title and description, is suitable for analysis due to its conveyance of purpose and content. In deciding whether the book was misleading or not, we relied upon external lists and signifiers of alternative cancer treatments. We sought to mitigate this limitation by auditing misleading coding application decisions. Finally, as Amazon.com search results are personalized based on prior behavior, it is possible that recommendations or highlighted products may appear even more frequently than reported here for users who previously engaged with such products or those closely related to them.

### Future Research

Future research into cancer misinformation on Amazon could include a focus on 3 areas in seeking to assess the potential dangers of such books: best sellers, advertising, and impact. Amazon has several lists for general and specific types of cancer, each of which has its own “best sellers” list that evolves over time [55]. It is prudent to track the best-selling, cancer-related “information” books for sale on Amazon for misinformation. Second, this study did not examine paid advertising of books containing cancer misinformation. Future research should determine the extent of paid advertising involving misleading cancer cure books on Amazon. Finally, the impact of misleading cancer books on their readers could be evaluated using survey or focus group methods. Other data sources, such as the reviews of misleading cancer cure books available on Amazon, could also be worthy of study for accuracy, provenance, and potential manipulation, to determine whether and how book reviews disseminate cancer misinformation.

## Appendix

Multimedia Appendix 1 Standards for Reporting Qualitative Research checklist.

## Abbreviations

FDA: Food and Drug Administration

## Data Availability

The coded data and media files are available upon reasonable request to the corresponding author.
